# Autoantibodies and Clinical Correlations in Polish Systemic Sclerosis Patients: A Cross-Sectional Study

**DOI:** 10.3390/jcm12020657

**Published:** 2023-01-13

**Authors:** Paweł Żebryk, Piotr Przymuszała, Jan Krzysztof Nowak, Tomasz Piorunek, Tatiana Mularek-Kubzdela, Mariusz Puszczewicz

**Affiliations:** 1 Department of Medical Education, Poznan University of Medical Sciences, 60-806 Poznań, Poland; 2 Department of Pediatric Gastroenterology and Metabolic Diseases, Poznan University of Medical Sciences, 60-572 Poznań, Poland; 3 Department of Pulmonology, Allergology and Pulmonary Oncology, Poznan University of Medical Sciences, 60-569 Poznań, Poland; 4 1st Department of Cardiology, Poznan University of Medical Sciences, 61-848 Poznań, Poland; 5 Department of Rheumatology, Rehabilitation and Internal Medicine, Poznan University of Medical Sciences, 61-545 Poznań, Poland

**Keywords:** systemic sclerosis, autoantibodies, antibodies, antinuclear, scleroderma, systemic

## Abstract

We evaluated the prevalence of systemic sclerosis (SSc)-related autoantibodies and their clinical significance and compared the sensitivity of two line immunoblot assays on a prospective study group of 96 Polish SSc patients (ACR-EULAR 2013 criteria) whose sera were assessed by indirect immunofluorescence (HEp-2 and monkey liver) and line immunoblot assays: ANA Profile 3 and Systemic Sclerosis Profile by EUROIMMUN (Lübeck, Germany). Organ involvement was evaluated according to the EUSTAR Minimal Essential Data Set. The following autoantibodies’ prevalence was found: Scl-70 (36%), Ro-52 (28%), CENP-B (22%), CENP-A (20%), PM-Scl-75 (20%), PM-Scl-100 (14%), fibrillarin (7%), Th/To (7%), RNA polymerase III 11 kDa (5%), RNA polymerase III 155 kDa (3%), PDGFR (3%), NOR-90 (2%), and Ku (1%). Significant associations between the autoantibodies’ presence and organ involvement were found: ATA (dcSSc > lcSSc, less prevalent muscle weakness), Ro-52 (gangrene, DLCO < 60), CENP-B and A (lcSSc > dcSSc, normal CK), CENP-B (rarer digital ulcers and joint contractures), PM-Scl-100 and 75 (PM/SSc overlap, CK increase, muscle weakness, muscle atrophy), PM-Scl-100 (dcSSc unlikely), PM-Scl-75 (lung fibrosis), fibrillarin (muscle atrophy, proteinuria, conduction blocks, palpitations), Th/To (proteinuria, arthritis, muscle weakness, and rarer esophageal symptoms), RNA Polymerase III 11 kDa (arterial hypertension, renal crisis), RNA polymerase III 155 kDa (renal crisis), and PDGFR (dcSSc, tendon friction rubs). Additionally, the Systemic Sclerosis Profile was significantly more sensitive in detecting SSc-related autoantibodies than ANA Profile 3 (*p* = 0.002). In conclusion, individual autoantibodies associated with specific characteristics of SSc.

## 1. Introduction

Systemic sclerosis (SSc) is a rare autoimmune connective tissue disorder of unknown etiology. Its exact pathogenesis and pathomechanism are not yet fully understood, but microcirculation dysfunction, complex immunological derangements, and extensive progressive fibrosis of almost all organs of the human body seem to play a crucial role [1]. The disease can manifest itself in two forms: limited cutaneous systemic sclerosis (lcSSc), which is more benign and affects the skin of the face and body parts distal to elbows and knees, and diffuse cutaneous systemic sclerosis (dcSSc), which has a more aggressive and dynamic course and involves the skin of most of the body as well as internal organs to a greater degree than the limited form does [2]. The clinical diagnosis of the disease used to be based on the American Rheumatism Association (ARA, now the American College of Rheumatology, ACR) criteria from 1980 [3]. Since ARA criteria were highly specific but their sensitivity was low, especially in the case of patients afflicted with the limited form of the disease, Le Roy and Medsger proposed criteria for the classification of early systemic sclerosis, which increased sensitivity from 33% to 92% in comparison with that of ARA criteria [4]. They added capillaroscopy and systemic sclerosis-specific antibodies to the traditional criteria, which in turn allowed a vast portion of patients with lcSSc to be diagnosed. Among SSc-specific antibodies, they listed antibodies against centromeres, topoisomerase I, fibrillarin, PM-Scl, and RNA polymerase I and III [4]. Meanwhile, according to different studies, in 90–95% of patients with systemic sclerosis, anti-nuclear antibodies can be detected by the indirect immunofluorescence method. In 2013, a joint ACR–EULAR committee developed new classification criteria for SSc, incorporating made advances, including specific antibodies such as anti-topoisomerase I (ATA), anti-centromere (ACA), and anti-RNA polymerase III (RNAP3). The committee also recommended that, when available, other antibodies (e.g., anti-Th/To, anti-U3 RNP) can be used as well.

It is worth noting that anti-topoisomerase I and anti-centromere antibodies can be detected in roughly 60% of systemic sclerosis patients. As a result, in 40% of patients meeting classification criteria, no autoantibodies were detected until recently, even though most of them showed presence of ANA in indirect immunofluorescence [5,6]. Although rarer antibodies were identified (against RNA Polymerase III, fibrillarin (U3-RNP), NOR90 (hUBF), Th/To, PM-Scl, Ku, and PDGFR), they could not be routinely detected in clinical practice due to difficult or unavailable methods (e.g., radioisotope method). However, recent progress in serology resulted in reliable, repeatable, and accessible tests that can be used for their detection [6,7,8,9]. This has been achieved using line immunoblot assays that exhibit excellent agreement with the gold standard (immunoprecipitation) [10]. The clinical importance of infrequently occurring SSc-related antibodies is less established than in the case of ATA or ACA. There are reports on a phenotypic variation of SSc-specific antibodies among patients from different ethnic groups, and there is a paucity of data on the Polish population [11]. Moreover, the retrospective design of previous investigations precluded an extended assessment of organ involvement and therefore a more detailed analysis of the clinical context of autoantibody presence.

Thus, in this study, we aimed to evaluate the prevalence of SSc-associated autoantibodies in a population of Polish patients with systemic sclerosis and to examine associations between their presence and a broad panel of clinical signs and symptoms of the disease. A secondary objective was to compare the sensitivity of the two line immunoblot assays: ANA Profile 3 (ANAP) and Systemic Sclerosis Profile (SSP).

## 2. Materials and Methods

The study was cross-sectional and prospective in design. It was carried out between 2012 and 2020 within the population of outpatients and inpatients of the Department of Rheumatology and Internal Medicine in Poznan, Poland. The study population consisted of 96 adult patients with SSc, classified using the American College of Rheumatology and the European League Against Rheumatism (ACR-EULAR) 2013 criteria [12]. There were no exclusion criteria.

History taking and physical examination of all patients were carried out by the same physician (P.Ż.), who has experience in the care of SSc patients. Clinical data were collected using MEDS (Minimal Essential Data Set) in line with the EUSTAR (EULAR Scleroderma Trials and Research group) methodology [13], also by the same practitioner. The following features were assessed: gender, age, time of disease since the onset of Raynaud’s phenomenon, time of disease since the onset of other symptoms characteristic of SSc, duration of the disease since the diagnosis, skin involvement using the modified Rodnan skin score (mRSS), Raynaud’s phenomenon, digital ulcerations, synovitis, joint contractures, muscle weakness, esophageal symptoms (heartburn and dysphagia), intestinal manifestations (diarrhea, constipation, and bloating), arterial hypertension, scleroderma renal crisis (ever), dyspnea, and palpitations.

Specialist cardiac (echocardiography) and pulmonary examination (pulmonary function tests and high-resolution computed tomography) were carried out. Certified specialist physicians interpreted the results: a cardiologist (T.M.-K.) and a pulmonologist (T.P.), both with considerable experience in SSc diagnostics. A number of characteristics were sought: heart conduction blocks; palpitations, systolic and diastolic dysfunction; ejection fraction; pulmonary arterial hypertension using Doppler ultrasonography; total lung capacity; pulmonary restriction using pulmonary function tests; and the presence of SSc-related interstitial lung disease (ILD) using HRCT.

Sera of patients were secured and assessed for autoantibodies. An immunoblot test known as Systemic Sclerosis Profile (SSP) by EUROIMMUN Medizinische Labordiagnostika AG (Lübeck, Germany) was used to assess SSc-related autoantibodies (Scl-70, centromere protein A, centromere protein B, RNA Pol III 11 kDa, RNA Pol III 155 kDa, fibrillarin, Nor90, Th/To, PM-Scl100, PM-Scl75, Ku, PDGFR, and Ro-52) according to the manufacturer’s recommendations, including the cutoff for seropositivity. Similarly, we used an ANA profile immunoblot by the same manufacturer to detect Scl-70, CENP-B, and PM-Scl. In addition, we determined the erythrocyte sedimentation rate (ESR), C-reactive protein concentration, serum creatinine concentration, the estimated glomerular filtration rate (chronic kidney disease epidemiology collaboration equation; eGFR CKD), as well as the presence or absence of proteinuria.

Statistical analyses were conducted in Statistica 13.3 (TIBCO, Palo Alto, CA, USA) and Microsoft Excel 2016 (Redmond, WA, USA). The associations between autoantibody seropositivity and clinical manifestations or disease subset were assessed using Fisher’s exact test, whereas the comparison of the sensitivity of both immunoblots was made using McNemar’s chi-square test. The significance threshold was set at 0.05. In cases where the contingency table included at least one zero while calculating odds ratios, we used Haldane–Anscombe correction by adding 0.5 to all values in a table so that calculating OR was mathematically possible. Heatmap was created in R 4.1.3 (R Software Foundation, Vienna, Austria) using the pheatmap package 1.0.12, with default hierarchical clustering, after logarithmic transformation (base 10) of odds ratios.

This study obtained the approval of the Bioethics Committee at the Poznan University of Medical Sciences (No. 988/11). All patients participating in the study gave informed consent in writing in line with local law regulations.

## 3. Results

### 3.1. Population Characteristics

**Figure 1:**
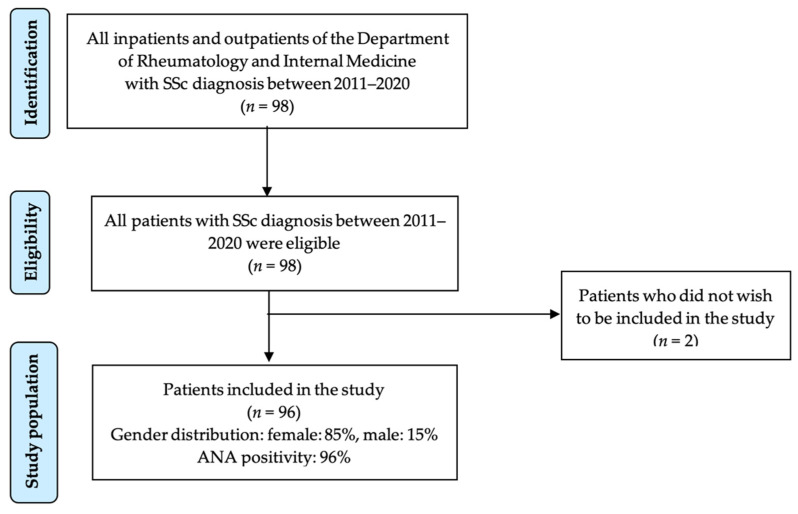
Enrollment of patients to the study.

Ninety-six patients meeting ACR-EULAR 2013 SSc classification criteria were included in the study, 56% of whom presented with limited cutaneous SSc, a third with diffuse cutaneous SSc, 9% with overlap syndromes and 1% as SSc sine scleroderma. Most patients were female and were included 6.2 ± 7.9 years after diagnosis of SSc. The patients were recruited from both outpatient and inpatient settings, as illustrated on a flowchart in Figure 1.

A detailed clinical and laboratory characteristic of the population studied is presented in Table 1.

### 3.2. SSc Specific Autoantibodies and Clinical Associations in the Studied Population

We assessed sera of patients included in the study for SSc-related antibodies using immunoblot and indirect immunofluorescence (IIF). Antinuclear antibodies by IIF were positive in 96% of patients with a median titer of 1/1280 (IQR 1/320-5120). We found the following prevalence of autoantibodies against antigens: Scl-70 (36%), Ro-52 (28%), CENP-B (22%), CENP-A (20%), PM-Scl-75 (20%), PM-Scl-100 (14%), fibrillarin (7%), Th/To (7%), RNA polymerase III 11 kDa (5%), RNA polymerase III 155 kDa (3%), PDGFR (3%), NOR-90 (2%), and Ku (1%).

**Figure 2:**
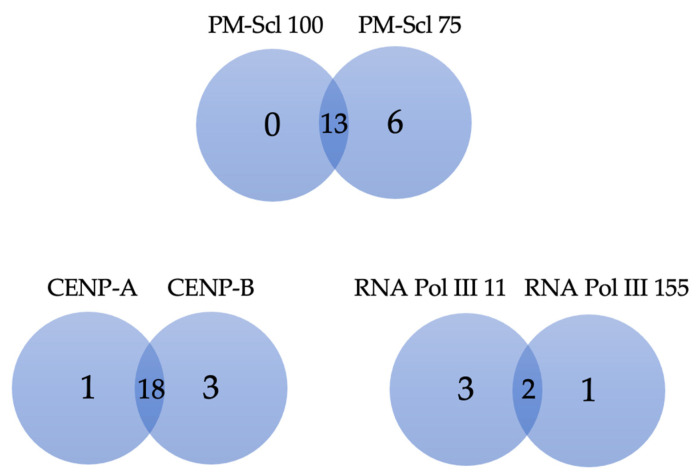
Venn’s diagram of SSc-related autoantibodies that have two variants of a target antigen. Numbers indicate how many patients’ sera reacted positively with specified target antigens.

The SSP immunoblot includes three cases where there are two antigen variants: PM-Scl, CENP, and RNA Polymerase. Figure 2 shows the numbers of patients positive for each of these autoantibody variants.

The analysis of thirty clinical features of SSc with all thirteen autoantibodies in the SSP found associations presented in Table 2, Table 3 and Table 4.

In our study, patients with ATA had dcSSc more often than lcSSc and less commonly presented with muscle weakness. By contrast, patients with either CENP-A or CENP-B antibodies presented with lcSSc disease form, while dcSSc was occurring less often and typically showed no CK elevation. Patients with CENP-B had fewer digital ulcers and joint contractures, and those with Ro-52 had a higher risk of gangrene and DLCO < 60%. No other significant associations were found, as shown in Table 2.

RNA Polymerase III 11 kDa positive patients had arterial hypertension more often, whereas both RNA Polymerase III 11 kDa and 150 kDa positive individuals had a high risk of renal crisis. Patients with anti-fibrillarin antibodies more frequently had muscle atrophy, proteinuria, conduction blocks, and palpitations. There were no associations in NOR90-positive patients, as presented in Table 3.

Patients positive for either PM-Scl-100 and -75 antibodies had a strong association with PM/SSc overlap, CK elevation, muscle weakness and muscle atrophy. In addition, those with PM-Scl-100 were unlikely to have the dcSSc disease subset, and those with PM-Scl-75 had higher odds of lung fibrosis by HRCT. Patients with Th/To antibody more often developed arthritis, muscle weakness, and proteinuria and had fewer esophageal symptoms than patients without this antibody. PDGFR-positive patients had more likelihood to have dcSSc subset and tendon friction rubs. No other significant associations were found, as shown in Table 4.

**Figure 3:**
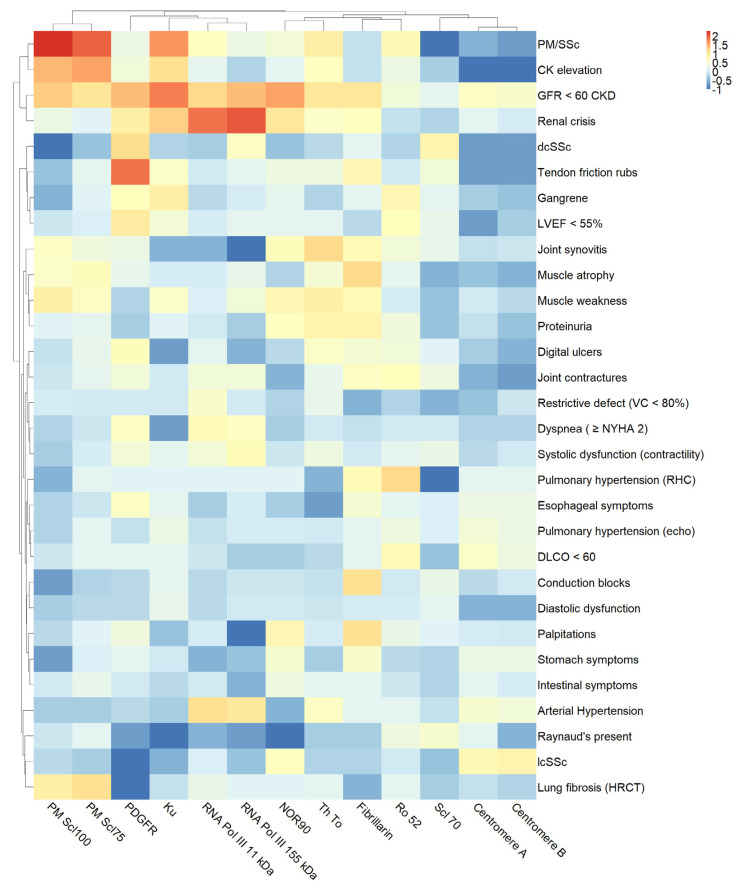
Heatmap illustrating odds ratios (ORs) for associations of selected SSc characteristics and autoantibodies. ORs were transformed logarithmically (base 10). Therefore, ORs < 1 are shown in blue, while ORs > 1 are in shades of red. Hierarchical clustering was used.

The odds ratios of the presence of clinical features of SSc in patients with positive reactions to SSc-related autoantibodies are visualized in Figure 3 as a heatmap.

### 3.3. Sensitivity of Systemic Sclerosis Profile vs. ANA Profile

To evaluate the sensitivity of SSP and ANAP we added all patients with a positive SSc-related autoantibody from each immunoblot and analyzed them in the contingency table. In SSP, patients positive for any antibodies from the list (Scl-70, CENP-B, CENP-A, PM-Scl-75, PM-Scl-100, fibrillarin, Th/To, RNA polymerase III 11 kDa, RNA polymerase III 155 kDa, PDGFR, NOR-90, and Ku) were included in the analysis, and similarly in ANAP (Scl-70, CENP-B and PM-Scl). We found that SSP detected SSc-related antibodies with significantly higher sensitivity (Mc Nemar chi-square B/C *p* = 0.002), as shown in Table 5.

### 3.4. SSc-Patients Negative for ATA, ACA, and RNAP3 (Triple-Negative)

In our cohort, 36% (35/96) of patients tested negative for ATA, ACA, and RNAP3. Within this triple-negative group, 20 patients had at least one autoantibody identified by SSP (three patients positive only for Ro-52 and 17 with either PM-Scl-75, PM-Scl-100, fibrillarin, Th/To, PDGFR, NOR-90, or Ku). Consequently, 16% (15/96) of patients in our study were negative for all 13 reactivities in SSP, and of these, only two were negative in the indirect immunofluorescence ANA test.

## 4. Discussion

Results of available studies on associations between SSc-specific antibodies and clinical symptoms of the disease conducted so far show many differences in the evaluation of organ involvement, antibodies detection, and inclusion criteria used (ACR, and/or LeRoy Medsger, ACR-EULAR 2013) [3,4,12]. This makes comparing and interpreting results more difficult and may be partially responsible for divergences between them. This study describes a number of relationships between SSc autoantibodies and its clinical manifestations, which is enhanced by the prospective design that allowed for systematic and uniform application of EUSTAR methodology of clinical assessment. Below we discuss these new findings in the context of the available literature.

### 4.1. Anti-Topoisomerase I (Anti-Scl-70) Antibodies (ATA)

The prevalence of ATA antibodies observed in this study lies within the literature range of 18–51% [7,8,14,15,16,17,18,19,20,21,22,23]. We found a strong correlation of ATA with the dcSSc form and a weaker negative correlation with the lcSSc subset, consistent with previous studies [5,15,18,22,24,25,26]. Interestingly, the sole association with organ symptoms in ATA-positive patients was the rarer occurrence of muscle weakness, which, to our best knowledge, has not been reported before. We did not see the commonly reported associations with SSc-ILD, PAH, and digital ulcers. Many studies compared patients with ATA vs. ACA or disease subsets like dcSSc vs. lcSSc and not, as in this study, ATA positive vs. ATA negative patients, and that can explain the differences [1,15,25]. Additionally, we used HRCT to detect SSc-ILD, and some studies relied on classical X-ray images, which show interstitial changes only in advanced disease [27,28]. Many patients have changes limited to approximately 13% of the lung parenchyma, which can be detected in HRCT but not in X-ray images [27].

### 4.2. Anti-Centromere Antibodies (ACA)

Our study’s percentage of ACA-positive patients falls within the reported range of 15–43% [7,8,14,15,16,17,20,21,22,23,29]. ACA-positive patients in our group had a more frequent occurrence of the lcSSc form and a less frequent dcSSc subset, consistent with the literature [30,31,32]. Patients with anti-CENP-B had a negative correlation with digital ulcers, joint contractures, and CK-elevation, but those positive for anti-CENP-A only with the latter.

### 4.3. Anti-RNA Polymerase III Antibodies (RNAP3)

This study’s prevalence of RNAP3 lies within the literature range from 1–15% [8,17,19,20,21,22,23,33,34,35,36,37]. RNAP3 correlated with arterial hypertension and renal crisis in our study group, consistent with other reports [33,34,35,36,38]. This is important since the high risk of renal crisis in RNAP3-positive patients necessitates frequent monitoring of blood pressure and creatinine. We found no associations to either lcSSc or dcSSc, but it can be explained by the relatively short time from diagnosis (1 to 3 years), as dcSSc can initially meet the criteria of lcSSc [4].

### 4.4. Anti-Th/To Antibodies

Anti-Th/To antibodies prevalence in this study was in the upper limit of the literature range of 0–7% [7,8,14,15,16,19,20,21,39,40,41]. There are reports of associations with SSc-ILD, SSc-PAH, and lcSSc [40,42]. Contrary to literature reports on the frequent occurrence of lcSSc in anti-Th/To positive patients [8,40,42,43], in this study, as in the study of Hamaguchi et al. [41], no significant connection with either form of the disease was observed, but such a trend is noticeable. In our cohort, anti-Th/To antibodies correlated with proteinuria, joint arthritis, muscle weakness, and rarer esophageal symptoms. Most studies to date did not collect data according to EUSTAR recommendations, which could explain no reports of such associations. Nevertheless, a recent report from Japan describes a case of an anti-Th/To antibody-positive patient with muscle weakness and necrotizing myopathy [44]. We found no associations of anti-Th/To antibodies with SSc-ILD and SSc-PAH in this study, which mirrors observations of Villalta and Gliddon [8,43].

### 4.5. Anti-Fibrillarin Antibodies (Anti-U3RNP, AFA)

The prevalence of AFA in this study constituted one of the higher results observed among the Caucasian race. Mehra et al. reported their occurrence among SSc patients in a range of 4–10% [15], while other researchers estimated it as 0–4% among Caucasian and Asian patients [7,8,14,41,45]. Interestingly, researchers [7,8,14,15] who used the same immunoblot kit (SSP) to detect antibodies showed the prevalence of AFA between 0.0 and 1.4%. Like others [45,46,47], our study found no correlation between AFA presence and dcSSc described elsewhere [16,41,48]. However, this may result from coexisting antibodies: six of seven AFA-positive patients had accompanying antibodies, in line with the report by Mehra et al. [15]. In this study, we found a previously unreported correlation with palpitations or conduction blocks, but since few studies assessed these parameters, we shall see if this corroborates in other cohorts. The only reference to heart involvement was a higher frequency of pericarditis in the Afro-American population with AFA reported by Sharif et al. [48]. We also found a more frequent occurrence of muscle atrophy, mirroring the results of other groups [15,46,47]. Tormey et al. [45] reported a link with renal involvement, which supports a correlation with proteinuria observed in this study and warrants regular follow-up of kidney function in AFA-positive patients.

### 4.6. Anti-PM-Scl Antibodies

The prevalence of PM-Scl-75 and anti-PM-Scl-100 in our study group was slightly higher than previously reported 0–13% [7,8,14,15,16,17,39,49]. In this study, anti-PM-Scl-75 and anti-PM-Scl-100 antibodies strongly correlated with PM/SSc overlap syndrome, CK elevation, muscle weakness, and muscle atrophy. These findings are consistent with the literature describing the involvement of muscles and PM/SSc overlap [49,50,51]. Patients with anti-PM-Scl-75 also had more frequent SSc-ILD than those negative for this reactivity, whereas anti-PM-Scl-100 correlated with the rarer occurrence of the dcSSc subset, confirming the findings of a study by Wodkowski et al., who found that anti-PM-Scl-75 patients had better survival than other antibody subsets [52]. Our study implies that patients with anti-PM-Scl-75 should have more frequent HRCT scans to check for SSc-ILD.

### 4.7. Anti-Ku Antibodies

Anti-Ku antibodies’ prevalence identified in this study was in the lower range of that in the literature: 1–5% [8,13,14,19,20,21,53]. They were detected in a female patient with polymyositis-scleroderma (PM/SSc) overlap syndrome and coexisted with anti-PM-Scl-100 and anti-PM-Scl-75 antibodies. The literature is scarce on anti-Ku phenotype, but it is associated with PM/SSc overlap and musculoskeletal system involvement, regardless of gender and diagnosis [53,54,55,56]. Thus, checking for myositis signs and symptoms and creatinine kinase in anti-Ku-positive patients seems essential.

### 4.8. Anti-NOR90 (Anti-hUBF) Antibodies

In this study, the prevalence of anti-NOR90 antibodies was 2% (2/96) compared to the literature range of 0–6% [8,57,58], and both patients had lcSSc. However, we found no correlation with the SSc subset nor organ involvement, which may be due to a few cases. The literature on anti-NOR90 is not robust, and their clinical relevance remains unclear [59], but they tend to be associated with lcSSc [8,16,59] and a better prognosis [60,61]. Some studies show no organ involvement [16], whereas others show an association with SSc-ILD [58,59]. Some reports link anti-NOR90-positive patients with cancer, but it certainly needs further research [57,59].

### 4.9. Anti-PDGFR Antibodies

The prevalence of anti-PDGFR antibodies was 3%, higher than previously reported 0–1% [8,15,58]. We found a correlation between anti-PDGFR antibodies and dcSSc, as previously reported. However, in all three cases, an additional antibody (ATA or AFA) was present, independently associated with this form of SSc. Furthermore, the only correlation of anti-PDGFR antibodies with organ manifestations in this study was tendon friction rubs. Nevertheless, due to the small number of patients, the authors were cautious in drawing conclusions, similarly to other researchers [7,8,14].

### 4.10. Anti-Ro-52 Antibodies

Our study’s prevalence of anti-Ro-52 antibodies is consistent with those reported previously: total 18–35% [8,15,16,20,22,62,63] and as isolated antibodies 0–6% [15,16]. No correlation with either form of SSc has been found so far [63], as in this study. However, the presented research demonstrates a correlation of anti-Ro-52 with gangrene and lowered DLCO < 60%. On the other hand, its correlations with SSc-ILD and overlap syndromes were not confirmed [62], but a positive association with PM/SSc, even though not statistically significant, was observed (*p* = 0.055).

### 4.11. Clinical Usefulness of Systemic Sclerosis Profile (SSP)

To the authors’ best knowledge, this is the first comparison of sensitivities of SSP and ANA Profile 3 in an SSc cohort. The sensitivity of SSP obtained is consistent with the range of 71–85% reported previously by other authors [7,8,14]. Here, we demonstrated SSP’s usefulness in detecting SSc-related antibodies in triple-negative patients (negative for ATA, ACA, and RNAP), a term used by Kruzer et al. [64]. Their recent study concluded that triple-negative SSc patients should undergo testing with an immunoblot assay like SSP because they have a higher risk of SSc-ILD and myopathy. A high level of agreement with reference methods, the simplicity of performance, and low cost [7,10] are arguments in favor of the routine use of SSP in antibody detection in SSc in clinical practice. The role of SSc-related autoantibodies beyond ATA and ACA still requires further research in multicenter studies with a standardized and comprehensive methodology like EUSTAR.

### 4.12. Limitations

Since this was a monocentric study, the study group may only be partially representative of the population of Polish SSc patients. The first author collaborated with outpatient rheumatology, dermatology, pulmonology, and cardiology clinics to increase the study group’s size by more than half. Extrapolating results on a broader population of SSc patients should be cautious. The study group involved 96 subjects, which is comparable to most studies published so far, given the low prevalence of SSc. Moreover, this study assessed the prevalence of very rare antibodies, and the number of positive patients can sometimes be low. Similarly, some organ manifestations of SSc, like a renal crisis, were present in just a single patient. That is why we used Fisher’s exact test, which is appropriate for small sample sizes. Furthermore, the main researcher performed a clinical examination of every patient, eliminating potential differences between researchers, but on the other hand, a systematic error could have occurred. Last but not least, given that the study did not include a control group, researchers could only estimate the sensitivity of methods for detecting antibodies without their specificity. On the other hand, among the strengths of the study, it should be noticed that it examined a broad set of potential clinical associations, noticeably larger than in most previous studies. This allowed to uncover some associations that were not previously described in the literature. Moreover, it involved not only so-called prototypical SSc antibodies (ATA, ACA, and RNAP3), but also others, less-commonly assessed, which is important given the still limited knowledge on their clinical associations. Additionally, in our study, we used comprehensive EUSTAR methods of data collection—MEDS. This should facilitate future meta-analyses, which will be indispensable to provide clear answers to questions regarding the clinical value of SSc autoantibodies.

## 5. Conclusions

The prevalence of antibodies in the examined population of SSc patients was: ATA (36%), Ro-52 (28%), PM-Scl-75 (20%), CENP-B (22%), CENP-A (20%), PM-Scl-100 (14%), fibrillarin (7%), Th/To (7%), RNA polymerase III 11 kDa (5%), PDGFR (3%), NOR-90 (2%), RNA polymerase III 155 kDa (3%), and Ku (1%). Correlations between the presence of antibodies and organ involvement were also found, which seems to allow for the identification of serological groups of patients with specific clinical characteristics: ATA (dcSSc > lcSSc, rarer muscle weakness), Ro-52 (gangrene, DLCO < 60), CENP-B and A (lcSSc > dcSSc, rare CK-elevation), CENP-B (rarer digital ulcers and joint contractures), PM-Scl-100 and 75 (PM/SSc overlap, CK increase, muscle weakness, muscle atrophy), PM-Scl-100 (rarer dcSSc), PM-Scl-75 (lung fibrosis), fibrillarin (muscle atrophy, proteinuria, palpitations and conduction blocks), Th/To (proteinuria, muscle weakness, joint synovitis and rarer esophageal symptoms), RNA Polymerase III 11 kDa (systemic hypertension, scleroderma renal crisis), RNA polymerase III 155 kDa (scleroderma renal crisis), and PDGFR (dcSSc, tendon friction rubs). Given the association of antibodies with specific clinical manifestations, it seems reasonable to promote their more frequent assessment. Individual antibodies may potentially serve as predictors of organ involvement and help in its monitoring and therapy. However, further research is needed to confirm the results of this and previous studies on clinical associations of SSc antibodies. Moreover, the detection of SSc-related antibodies with Systemic Sclerosis Profile significantly improves the sensitivity of serological classification compared to that with ANA Profile 3, which can be helpful in the early stages of the disease.

## Figures and Tables

**Table 1:** Study population characteristics, *n* = 96. Data are presented as percentage or mean (SD).

Variable	Mean (or %)	SD
Gender, female, %	85%	
Limited cutaneous systemic sclerosis	56%	
Diffuse cutaneous systemic sclerosis	33%	
Polymyositis-scleroderma overlap syndrome	7%	
Systemic sclerosis sine scleroderma	1%	
Age, years	53.7	13.8
Disease duration since Raynaud, years	11.3	8.9
Disease duration since diagnosis, years ^1^	6.2	7.9
ESR, mm/h	18.8	15.8
CRP, mg/L	4.4	9.3
Serum creatinine, umol/L	86.3	25.8
eGFR CKD EPI, mL/min/1.73 m^2^	91.9	13.6
mRSS score ^2^	12.4	8.2
Raynaud’s present, %	97%	
Lung fibrosis (HRCT), %	69%	
Joint contractures, %	63%	
Esophageal symptoms (dysphagia, reflux), %	60%	
Dyspnea (≥NYHA 2), %	58%	
Digital ulcers, %	52%	
Joint synovitis, %	44%	
Muscle weakness, %	42%	
Arterial hypertension, %	40%	
Palpitations, %	38%	
Intestinal symptoms (diarrhea, bloating, constipation), %	26%	
Gastric symptoms (early satiety, vomiting), %	23%	
Muscle atrophy, %	21%	
Proteinuria, %	19%	
CK-elevation, %	17%	
Restrictive defect (VC < 80%) (*n* = 64), %	16%	
Systolic dysfunction (contractility), %	15%	
Conduction blocks, %	15%	
Diastolic dysfunction, %	14%	
Pulmonary hypertension (echocardiography), %	13%	
Gangrene, %	9%	
Tendon friction rubs, %	7%	
Renal crisis, %	1%	
eGFR CKD EPI < 60 mL/min/1.73 m^2^	1%	
FVC (%predicted) (*n* = 49), %	88.9	22.5
TLC (%predicted) (*n* = 60), %	96.9	24.7
LVEF, %	61.3	9.2

^1^ Disease duration calculated since the onset of first non-Raynaud phenomenon features of disease. ^2^ The median modified Rodnan skin score was 11 (1st–3rd quartile: 7–16). SD = standard deviation, eGFR CKD-EPI—estimated glomerular filtration rate CKD-EPI equation, CRP—C-reactive protein, ESR—erythrocyte sedimentation rate, FVC—forced vital capacity, TLC—total lung capacity, LVEF—left-ventricular ejection fraction, mRSS—modified Rodnan skin score, NYHA—New York Heart Association (NYHA) Functional Classification of heart failure.

**Table 2:** Analysis of associations between clinical features of SSc and autoantibodies using Fisher’s exact test and OR (95% CI).

	Scl-70		Centromere A		Centromere B		Ro-52	
	*p*	OR (95% CI)	*p*	OR (95% CI)	*p*	OR (95% CI)	*p*	OR (95% CI)
lcSSc	0.019	0.4 (0.1–0.8)	0.009	5.5 (1.5–20.4)	0.002	6.5 (1.8–24)	1.000	1 (0.4–2.3)
dcSSc	0.000	6.4 (2.5–16.4)	0.016	0.2 (0–0.8)	0.008	0.2 (0–0.7)	0.343	0.6 (0.2–1.6)
PM/SSc	0.080	0.1 (0–2.1)	0.342	0.3 (0–5.1)	0.332	0.2 (0–4.5)	0.055	5.7 (1–32.9)
Raynaud’s present	0.530	3 (0.1–64.8)	1.000	1.4 (0.1–30)	0.410	0.3 (0–4.8)	1.000	2.2 (0.1–47.4)
Digital ulcers	0.648	1.3 (0.5–3.2)	0.270	0.5 (0.2–1.5)	0.040	0.3 (0.1–0.9)	0.090	2.5 (0.9–7.3)
Gangrene	0.723	1.4 (0.3–5.5)	0.681	0.5 (0.1–4.1)	0.677	0.4 (0–3.5)	0.016	6.1 (1.4–26.8)
Joint synovitis	0.361	1.7 (0.7–4.2)	0.778	0.8 (0.3–2.4)	1.000	0.9 (0.3–2.6)	0.096	2.4 (0.9–6.4)
Joint contractures	0.287	2.1 (0.7–6.5)	0.060	0.3 (0.1–1)	0.014	0.2 (0.1–0.7)	0.080	4.4 (0.9–20.8)
Tendon friction rubs	0.419	2.4 (0.5–11.2)	0.338	0.2 (0–4.4)	0.340	0.2 (0–3.8)	1.000	1 (0.2–5.5)
CK-elevation	0.389	0.5 (0.2–1.8)	0.034	0.1 (0–1.7)	0.019	0.1 (0–1.5)	0.344	1.9 (0.6–6.2)
Muscle weakness	0.040	0.4 (0.1–0.9)	1.000	1 (0.3–3.2)	0.598	0.7 (0.2–2)	1.000	1.1 (0.4–2.9)
Muscle atrophy	0.055	0.3 (0.1–1)	0.506	0.4 (0.1–2.1)	0.217	0.3 (0.1–1.6)	0.395	1.6 (0.6–4.9)
Esophageal symptoms (dysphagia, reflux)	0.815	1.2 (0.5–3)	0.277	2 (0.6–6.9)	0.299	1.9 (0.6–5.9)	0.629	1.4 (0.5–3.6)
Stomach symptoms (early satiety, vomiting)	0.443	0.6 (0.2–1.7)	0.350	1.9 (0.6–5.9)	0.253	1.9 (0.6–5.6)	0.592	0.7 (0.2–2.1)
Intestinal symptoms (diarrhea, bloating, constipation)	0.324	0.6 (0.2–1.6)	0.545	1.5 (0.5–4.8)	1.000	1.1 (0.3–3.2)	1.000	0.9 (0.3–2.6)
Arterial Hypertension	0.652	0.8 (0.3–1.8)	0.055	3.1 (1–9.4)	0.121	2.5 (0.9–7)	0.633	1.4 (0.5–3.5)
Dyspnea (≥NYHA 2)	1.000	1 (0.4–2.5)	0.392	0.6 (0.2–1.8)	0.422	0.6 (0.2–1.8)	1.000	1 (0.4–2.6)
Palpitations	0.635	1.3 (0.5–3.4)	1.000	1.1 (0.4–3.6)	1.000	1 (0.3–2.9)	0.305	1.8 (0.6–5)
Conduction blocks	0.372	1.8 (0.6–5.7)	1.000	0.7 (0.1–3.3)	1.000	1 (0.2–4)	1.000	1 (0.3–3.6)
Diastolic dysfunction	0.546	1.5 (0.5–4.9)	0.446	0.3 (0–2.6)	0.281	0.3 (0–2.2)	1.000	1.1 (0.3–4.1)
LVEF < 55%	0.476	1.7 (0.4–7.3)	0.338	0.2 (0–4.2)	0.678	0.5 (0.1–4.2)	0.098	4.5 (1–20.6)
Systolic dysfunction (contractility)	0.376	1.8 (0.6–5.6)	0.728	0.7 (0.1–3.3)	1.000	1 (0.2–4)	0.518	1.6 (0.5–5.3)
Pulmonary hypertension (ECHO)	1.000	1.2 (0.3–4)	0.216	2.7 (0.7–10.6)	0.282	2 (0.5–7.7)	0.299	2.1 (0.6–7.5)
Pulmonary hypertension (RHC)	0.143	0.1 (0–2.1)	1.000	1.5 (0.1–40.6)	1.000	1.5 (0.1–40.6)	0.143	16.3 (0.5–555.7)
Restrictive defect (VC < 80%)	0.074	0.3 (0.1–1.1)	0.667	0.4 (0–3.7)	1.000	0.9 (0.2–4.9)	0.740	0.6 (0.1–2.5)
Lung fibrosis (HRCT)	0.171	0.5 (0.2–1.4)	0.714	0.8 (0.2–3.3)	0.481	0.6 (0.2–2.3)	0.765	1.4 (0.4–5)
DLCO < 60%	0.105	0.4 (0.1–1.2)	0.418	3.7 (0.4–32.3)	0.481	2.1 (0.4–10.7)	0.036	5.3 (1.1–26.1)
Proteinuria	0.176	0.4 (0.1–1.3)	1.000	0.8 (0.2–3.1)	0.342	0.4 (0.1–1.9)	0.150	2.3 (0.8–6.7)
eGFR CKD EPI < 60 mL/min/1.73 m^2^	1.000	1.6 (0–84.9)	1.000	3.9 (0.1–201.4)	1.000	3.4 (0.1–177.4)	1.000	2.5 (0–126.8)
Renal crisis	1.000	0.6 (0–14)	1.000	1.4 (0.1–35.4)	1.000	1.1 (0–28.8)	1.000	0.8 (0–20.2)

**Table 3:** Analysis of associations between clinical features of SSc and autoantibodies using Fisher’s exact test and OR (95% CI).

	RNA Pol III 11 kDa		RNA Pol III 155 kDa		Fibrillarin		NOR90	
	*p*	OR (95% CI)	*p*	OR (95% CI)	*p*	OR (95% CI)	*p*	OR (95% CI)
lcSSc	1.000	1.2 (0.2–7.5)	0.581	0.4 (0–4.4)	0.695	0.6 (0.1–2.7)	0.502	4.1 (0.2–88)
dcSSc	0.657	0.5 (0–4.3)	0.271	4 (0.3–45.9)	0.689	1.5 (0.3–7)	0.544	0.4 (0–7.9)
PM/SSc	0.289	4.2 (0.4–44.4)	1.000	1.9 (0.1–40)	1.000	0.8 (0–16.1)	1.000	2.6 (0.1–60.7)
Raynaud’s present	1.000	0.3 (0–6.4)	1.000	0.2 (0–5.1)	1.000	0.5 (0–10.5)	1.000	0.1 (0–3.9)
Digital ulcers	1.000	1.4 (0.1–15.7)	0.562	0.3 (0–3.7)	0.643	2.8 (0.3–26.7)	1.000	0.7 (0–11)
Gangrene	1.000	0.7 (0–13.4)	1.000	1.1 (0.1–23.1)	0.557	1.5 (0.2–13.7)	1.000	1.6 (0.1–35.2)
Joint synovitis	0.348	0.3 (0–2.9)	0.108	0.1 (0–2.5)	0.203	5.1 (0.6–46)	0.495	4.9 (0.2–104.8)
Joint contractures	0.571	2.5 (0.1–49.5)	0.571	2.5 (0.1–49.5)	0.326	4 (0.2–75.4)	0.435	0.3 (0–5.3)
Tendon friction rubs	1.000	1 (0–18.9)	1.000	1.5 (0.1–32.6)	0.091	6.2 (1–40.6)	1.000	2.2 (0.1–49.6)
CK-elevation	0.552	1.6 (0.2–16.2)	1.000	0.6 (0–12.5)	1.000	0.8 (0.1–6.7)	1.000	1.5 (0.1–37.9)
Muscle weakness	1.000	1.2 (0.2–8.9)	0.591	2.4 (0.2–28.1)	0.174	5.2 (0.6–48.5)	0.207	6.2 (0.3–133.9)
Muscle atrophy	1.000	1.1 (0.1–11.5)	0.547	1.7 (0.1–20.1)	0.009	16.8 (1.7–161.4)	1.000	0.6 (0–13.9)
Esophageal symptoms (dysphagia, reflux)	0.608	0.5 (0.1–3.9)	1.000	1.1 (0.1–12.4)	0.660	2.8 (0.3–25.5)	1.000	0.5 (0–8.7)
Stomach symptoms (early satiety, vomiting)	0.568	0.3 (0–5.9)	0.568	0.4 (0–8)	0.167	3.3 (0.6–17.5)	0.444	3 (0.2–50.9)
Intestinal symptoms (diarrhea, bloating, constipation)	1.000	1 (0.1–12.1)	0.547	0.3 (0–5.6)	0.657	1.4 (0.2–9.1)	0.547	2.1 (0.1–35.4)
Arterial Hypertension	0.028	14 (0.7–268.5)	0.070	10.6 (0.5–210.9)	0.691	1.4 (0.3–7.5)	0.508	0.3 (0–5.7)
Dyspnea (≥NYHA 2)	0.294	5.1 (0.3–97.2)	0.548	3.9 (0.2–77.3)	1.000	0.8 (0.1–4.9)	1.000	0.5 (0–8.4)
Palpitations	1.000	1.1 (0.1–8.4)	0.242	0.1 (0–2.9)	0.020	14.1 (0.8–265.5)	0.221	5.9 (0.3–126.5)
Conduction blocks	1.000	0.7 (0–13.6)	1.000	0.9 (0–20.7)	0.006	13.6 (2.2–84.2)	1.000	0.9 (0–20.7)
Diastolic dysfunction	1.000	0.7 (0–14.9)	1.000	1 (0–22.7)	1.000	1.1 (0.1–10.3)	1.000	1 (0–22.7)
LVEF < 55%	1.000	1.1 (0.1–23.9)	1.000	1.6 (0.1–36.5)	1.000	0.7 (0–13.8)	1.000	1.6 (0.1–36.5)
Systolic dysfunction (contractility)	0.430	2.6 (0.2–30.6)	0.311	5.2 (0.3–89.1)	1.000	1 (0.1–9.1)	1.000	0.9 (0–20.4)
Pulmonary hypertension (ECHO)	1.000	0.8 (0–16.1)	1.000	1.1 (0.1–24.6)	0.552	1.5 (0.2–14.9)	1.000	1.1 (0.1–24.6)
Pulmonary hypertension (RHC)	1.000	1.3 (0–82.5)	1.000	1.3 (0–82.5)	0.429	5.4 (0.2–188.8)	1.000	1.3 (0–82.5)
Restrictive defect (VC < 80%)	0.422	3.4 (0.2–57.2)	1.000	1 (0–26.4)	0.564	0.3 (0–6.3)	1.000	0.6 (0–13.2)
Lung fibrosis (HRCT)	1.000	1.8 (0.1–37.6)	1.000	1.3 (0.1–28.4)	0.135	0.3 (0.1–1.4)	1.000	1.3 (0.1–28.4)
DLCO < 60	1.000	0.9 (0.1–10.9)	0.538	0.5 (0–7.6)	1.000	1.4 (0.1–14.6)	0.538	0.5 (0–7.6)
Proteinuria	1.000	1 (0.1–9.7)	1.000	0.5 (0–11)	0.026	6.7 (1.3–33.1)	0.358	4.2 (0.3–71.2)
eGFR CKD EPI < 60 mL/min/1.73 m^2^	1.000	16.1 (0.3–890.5)	1.000	25.9 (0.4–1503.3)	1.000	11.5 (0.2–623.7)	1.000	36.6 (0.6–2245)
Renal crisis	0.045	73.3 (2.5–2140.5)	0.034	103.8 (3.3–3233.3)	1.000	4.2 (0.2–114.6)	1.000	11.5 (0.4–359.3)

**Table 4:** Analysis of associations between clinical features of SSc and autoantibodies using Fisher’s exact test and OR (95% CI).

	Th/To		PM-Scl100		PM-Scl75		Ku		PDGFR	
	*p*	OR (95% CI)	*p*	OR (95% CI)	*p*	OR (95% CI)	*p*	OR (95% CI)	*p*	OR (95% CI)
lcSSc	0.695	0.6 (0.1–2.7)	0.554	0.7 (0.2–2.1)	0.209	0.5 (0.2–1.4)	0.441	0.3 (0–6.5)	0.082	0.1 (0–2.1)
dcSSc	1.000	0.7 (0.1–4.1)	0.004	0.1 (0–1)	0.278	0.4 (0.1–1.5)	1.000	0.6 (0–15.7)	0.038	14.6 (0.7–291.8)
PM/SSc	0.063	8.2 (1.2–56.1)	0.000	188.1 (9.6–3673.8)	0.000	90.6 (4.9–1687.7)	0.065	47.7 (1.7–1313.1)	1.000	1.9 (0.1–40)
Raynaud’s present	1.000	0.5 (0–10.5)	1.000	0.9 (0–19.4)	1.000	1.4 (0.1–30)	1.000	0.1 (0–2.7)	1.000	0.2 (0–5.1)
Digital ulcers	0.393	3.6 (0.4–32.6)	0.753	0.8 (0.2–2.9)	0.414	1.7 (0.5–5.4)	0.402	0.2 (0–5.5)	0.270	5 (0.3–100.9)
Gangrene	1.000	0.6 (0–11)	0.590	0.3 (0–6.3)	0.672	1.3 (0.2–6.9)	1.000	8.1 (0.2–429.9)	0.288	4.6 (0.4–56.8)
Joint synovitis	0.012	16.6 (0.9–301.7)	0.068	3.8 (1–15.1)	0.186	2.3 (0.8–6.7)	0.482	0.3 (0–7.6)	1.000	1.9 (0.2–21.8)
Joint contractures	1.000	1.7 (0.2–15.5)	1.000	0.9 (0.2–3.8)	0.750	1.6 (0.4–6.4)	1.000	1 (0–25.9)	0.571	2.5 (0.1–49.5)
Tendon friction rubs	0.444	2.1 (0.2–20.8)	0.587	0.4 (0–6.6)	0.633	1.6 (0.3–8.8)	1.000	3.7 (0.1–98.1)	0.005	75.9 (3.2–1783.2)
CK-elevation	0.104	4.1 (0.8–20.5)	0.000	28.3 (6.2–129.5)	0.000	38.4 (9–164.1)	0.179	14.4 (0.6–371)	0.450	2.4 (0.2–28.3)
Muscle weakness	0.044	8.3 (0.9–71.9)	0.005	8.4 (1.7–41)	0.017	4 (1.3–12.6)	0.458	3.6 (0.1–92)	1.000	0.6 (0.1–6.7)
Muscle atrophy	0.193	2.8 (0.6–13.9)	0.070	3.5 (1–12)	0.010	5 (1.6–15.5)	1.000	1.1 (0–27.7)	0.547	1.7 (0.1–20.1)
Esophageal symptoms (dysphagia, reflux)	0.047	0.2 (0–1)	0.358	0.6 (0.2–1.8)	0.792	0.9 (0.3–2.5)	1.000	1.6 (0.1–41.7)	0.549	4 (0.2–79.9)
Stomach symptoms (early satiety, vomiting)	0.673	0.5 (0.1–4.1)	0.281	0.2 (0–1.9)	0.768	1.2 (0.4–3.8)	1.000	1 (0–24.3)	1.000	1.5 (0.1–17.4)
Intestinal symptoms (diarrhea, bloating, constipation)	0.657	1.4 (0.2–9.1)	1.000	1 (0.2–4.6)	0.364	1.7 (0.5–5.7)	1.000	0.7 (0–17.1)	1.000	1 (0.1–12.1)
Arterial Hypertension	0.124	3.9 (0.7–21.2)	0.346	0.5 (0.1–1.9)	0.284	0.5 (0.2–1.6)	1.000	0.5 (0–11.4)	1.000	0.7 (0.1–7.9)
Dyspnea (≥NYHA 2)	1.000	1 (0.2–6)	0.499	0.6 (0.2–2.1)	1.000	0.9 (0.3–2.9)	0.341	0.2 (0–4.3)	0.548	3.9 (0.2–77.3)
Palpitations	1.000	1.1 (0.2–5.9)	0.740	0.7 (0.2–2.7)	0.774	1.3 (0.4–4.2)	1.000	0.4 (0–9.1)	0.601	2.3 (0.2–26.4)
Conduction blocks	1.000	0.8 (0.1–7.4)	0.197	0.2 (0–3.2)	0.725	0.6 (0.1–3)	1.000	1.6 (0.1–41.2)	1.000	0.7 (0–13.6)
Diastolic dysfunction	1.000	0.9 (0.1–8.2)	1.000	0.5 (0.1–4.4)	1.000	0.7 (0.1–3.4)	1.000	1.7 (0.1–45)	1.000	0.7 (0–14.9)
LVEF < 55%	0.546	1.5 (0.2–14.5)	1.000	0.9 (0.1–7.7)	1.000	1.2 (0.2–6.7)	1.000	2.7 (0.1–72.4)	0.196	9.9 (0.6–175.4)
Systolic dysfunction (contractility)	0.336	2.1 (0.4–12.3)	0.681	0.5 (0.1–3.9)	1.000	1.1 (0.3–4.4)	1.000	1.6 (0.1–40.6)	0.430	2.6 (0.2–30.6)
Pulmonary hypertension (ECHO)	1.000	1 (0.1–9)	1.000	0.6 (0.1–4.8)	0.704	1.4 (0.3–5.7)	1.000	1.9 (0.1–48.8)	1.000	0.8 (0–16.1)
Pulmonary hypertension (RHC)	1.000	0.3 (0–11.3)	1.000	0.3 (0–11.3)	1.000	1.5 (0.1–40.6)	1.000	1.3 (0–82.5)	1.000	1.3 (0–82.5)
Restrictive defect (VC < 80%)	0.622	1.7 (0.3–10.3)	1.000	1.1 (0.3–4.7)	1.000	1 (0.3–3.6)	1.000	1 (0–26.4)	1.000	1 (0–26.4)
Lung fibrosis (HRCT)	1.000	1.5 (0.2–13.7)	0.062	8.3 (0.5–147.8)	0.017	13.5 (0.8–237.1)	1.000	0.8 (0–19.7)	0.098	0.1 (0–1.3)
DLCO < 60	0.649	0.7 (0.1–4.4)	1.000	0.9 (0.2–3.5)	0.757	1.5 (0.4–5.6)	1.000	1.4 (0.1–37.1)	1.000	1.4 (0.1–37.1)
Proteinuria	0.026	6.7 (1.3–33.1)	0.714	1.3 (0.3–5.2)	0.516	1.6 (0.5–5.4)	1.000	1.3 (0.1–33.4)	1.000	0.5 (0–11)
eGFR CKD EPI < 60 mL/min/1.73 m^2^	1.000	11.5 (0.2–623.7)	0.138	19.6 (0.8–507.3)	0.202	12.2 (0.5–312.9)	1.000	61.7 (0.9–4275.8)	1.000	25.9 (0.4–1503.3)
Renal crisis	1.000	3.6 (0.1–96.9)	1.000	2 (0.1–52.9)	1.000	1.3 (0–32.5)	1.000	19.4 (0.5–700.7)	1.000	8.1 (0.3–237.8)

**Table 5:** The sensitivity analysis of Systemic Sclerosis Profile vs. ANA Profile 3rd generation by EUROIMMUN.

	ANAP+	ANAP−	
SSP + *n*, %	65	12	77
	68%	12%	80%
SSP − *n*, %	0	19	19
	0%	20%	20%
	65	31	96
	68%	32%	100%

## Data Availability

The data used to support the findings in this study are available from the corresponding author upon reasonable request.
